# Serum microRNA-21 levels are related to tumor size in gastric cancer patients but cannot predict prognosis

**DOI:** 10.3892/ol.2013.1626

**Published:** 2013-10-15

**Authors:** JIANNING SONG, ZHIGANG BAI, JUN ZHANG, HUA MENG, JUN CAI, WEI DENG, JINTAO BI, XUEMEI MA, ZHONGTAO ZHANG

**Affiliations:** Department of General Surgery, Beijing Friendship Hospital, Capital Medical University, Xuanwu, Beijing 100050, P.R. China

**Keywords:** biomarker, microRNA-21, stomach neoplasma, prognosis

## Abstract

In patients with gastric cancer (GC), circulating microRNA-21 (miR-21) is overexpressed and may serve as a diagnostic biomarker. In the present study, it was hypothesized that the serum miR-21 expression levels were associated with prognosis in the patients with GC. The expression levels of serum miR-21 were measured using quantitative polymerase chain reaction (qPCR) assays in 103 GC patients. Survival and Cox proportional-hazards regression analyses were performed to determine the correlation between serum miR-21 expression levels and prognosis in the patients. The correlation between the serum miR-21 levels and the clinicopathological factors of the patients was also analyzed. Survival curves were not significantly different between the groups exhibiting high and low levels of serum miR-21 expression. High levels of miR-21 in the serum were associated with an increased tumor size and an advanced pT stage. These findings suggest that serum miR-21 could be exploited as a practical biomarker for monitoring tumor burden in patients with GC.

## Introduction

Gastric cancer (GC) is one of the most common causes of mortality worldwide (1). In GC patients, survival and prognosis mainly depend on the TNM stage of the tumor at diagnosis. However, novel, specific, non-invasive biomarkers that are able to identify high-risk patients with a poor prognosis are urgently required. microRNAs (miRNAs) are single-stranded RNA molecules (21–23 nucleotides) that regulate gene expression by either interfering with transcription or inhibiting translation. These miRNAs play important roles in various human biological processes, including metabolism, differentiation, cell proliferation and apoptosis (2). Altered miRNA expression has been reported in various types of cancer, indicating that miRNAs may be involved in cancer tumorigenesis. A number of studies have shown that microRNAs circulate in the bloodstream in a highly stable, extracellular form (3,4) and may therefore be used as blood-based biomarkers for cancer and other diseases (5–7).

One of the most intensively studied miRNAs is miRNA-21 (miR-21). miRNA-21 has been shown to be overexpressed in numerous types of tumor tissues (8). It has been demonstrated that miR-21 is involved in cancer at almost all stages (9). In GC cell lines, miR-21 has been shown to promote proliferation and invasion, inhibit apoptosis and regulate cell migration (10,11). In clinical studies, miR-21 has been consistently overexpressed in GC tissues compared with corresponding normal gastric tissues (12). Tissue miR-21 levels have been reported to be significantly associated with differentiation, lymph node metastasis (11), tumor size, depth of invasion and TNM stage (13,14) in GC patients. Jiang *et al*(15) reported that miR-21 was significantly associated with S-1/oxaliplatin responses in GC patients. In serum/plasma samples from GC patients, miR-21 was also reported to be overexpressed and was considered to possibly serve as a diagnostic biomarker (16). Serum miR-21 has been reported to be significantly reduced after tumor resection in patients with GC, head and neck squamous cell carcinoma and lung carcinoma (17). In patients with diffuse large B-cell lymphoma, high serum miR-21 levels indicate a shorter relapse-free survival time (18). However, data concerning the possible prognostic role of serum miR-21 levels in GC are limited.

Based on previous data on miR-21, we propose that serum miR-21 may be related to the prognosis in GC. In the present study, the expression levels of serum miR-21 were detected in 103 GC cases using quantitative polymerase chain reaction (qPCR). Survival curves were compared using the log-rank test and Cox regression analysis to test the hypothesis that serum miR-21 levels were related to the prognosis in GC. Furthermore, the expression of serum miR-21 was analyzed and its correlation with the clinicopathological factors in GC was investigated.

## Materials and methods

### Patients and serum samples

Serum samples were obtained from 103 patients with GC that had been surgically treated in the Beijing Friendship Hospital (Beijing, China) between 2008 and 2009. No patients in this study had received chemotherapy or radiotherapy prior to blood sampling. Venous blood (5 ml) was collected from each patient prior to surgery and centrifuged at 125 × g for 10 min. Supernatants were recovered and stored at −80°C until further analysis. Follow-up data for all recruited patients were acquired and the survival time was calculated from the date of surgery to the date of mortality or last follow-up on 20 June, 2012. Written informed consent was obtained from each patient, and study approval was obtained from the Beijing Friendship Hospital ethics review board.

### RNA extraction and reverse transcription

RNA for serum/plasma samples was isolated using an miRcute miRNA isolation kit (Tiangen Biotech, Beijing, China), according to the manufacturer’s instructions, with some modifications. Briefly, 300 μl human serum was mixed with 300 μl lysis buffer. Subsequent to phase separation, the aqueous phase was mixed with ethanol then added to miRspin and miRelute columns. The microRNA was eluted with 30 μl RNase-free water, with 18 μl used for reverse transcription. The RNA concentration and purity were assessed using an Eppendorf BioPhotometer (Eppendorf, Hamburg, Germany). The RNA concentrations ranged from 26–54 ng/μl. The purity of RNA was verified by measuring the absorbance of the samples at 260 and 280 nm and determining the 260/280 ratio (acceptable range, 1.77–1.92).

Reverse transcription was carried out using an all-in-one miRNA first-strand cDNA synthesis kit (GeneCopoeia, Rockville, MD, USA). Final reaction volumes were 25 μl and contained 1 μl 2.5 U/μl poly-A polymerase, 1 μl RTase mix, 5 μl 5X reaction buffer and 18 μl purified miRNA. Reverse transcription was performed in a PTC-200 peltier thermal cycler (Bio-Rad Laboratories, Shanghai, China) at 37°C for 60 min and then 85°C for 5 min.

### Detection of serum miRNAs by qPCR

The qPCRs were conducted using an Applied Biosystems (ABI) 7500 thermal cycler (Invitrogen, Carlsbad, CA, USA) in 96-well plates. Each miRNA assay was performed in duplicate with a non-template control contained in each plate. To control for inter-assay variation, the samples analyzed on the same plate were for one specific miRNA. An all-in-one miRNA qPCR kit (GeneCopoeia) was used, with 20 μl qPCR mixtures containing 10 μl 2X all-in-one qPCR mix, 2 μl all-in-one miRNA qPCR primer, 2 μl universal adaptor primer, 0.4 μl 50X ROX reference dye and 5.6 μl cDNA. Amplification was performed on an ABI 7500 with a cycling profile of 50°C for 2 min and 95°C for 10 min, followed by 50 cycles of 95°C for 15 sec and 60°C for 1 min. At the end of the 50th cycle, a melt curve analysis was carried out to verify any non-specific amplification.

### Statistical analysis

The relative quantity (Qrel) of miR-21 was quantified using the comparative ΔCt method using the following equation: Qrel = E^^^ − (Cq_test sample_ − Cq_Average of miR-16 and miR-93_), where E^^^ is the power of the PCR amplification efficiency and Cq is the quantification cycle.

We have previously reported that miR-16 and miR-93 may serve as double reference genes for the qPCR analysis of serum miRNAs in GC samples (19). In the present study, the statistical analysis was performed using SPSS 17.0 (SPSS, Inc., Chicago, IL, USA). Fisher’s exact test was used to compare the difference between the serum miR-21 high and low expression groups. The correlation between overall survival and serum miR-21 was analyzed using the Kaplan-Meier method and the log-rank test. The Cox proportional-hazards regression analysis was used to evaluate whether serum miR-21 was an independent prognostic factor for GC. All statistical tests were two-sided and P<0.05 was considered to indicate a statistically significant difference.

## Results

### Clinical characteristics of study participants with GC

The clinical characteristics of the study participants are listed in Table I. The study cohort was comprised of 68 males and 35 females. The mean age was 60 years (range, 27–87 years) and the median follow-up period was 35.9 months (range, 24.4–53.1 months). Of these patients, 80 underwent radical surgery and 23 underwent palliative surgery. None of the patients received peri-operative chemotherapy. The patients were at TNM stage I (n=23), II (n=8), III (n=58) and IV (n=14). At the last follow-up on June 20, 2012, 53 patients were still alive.

### Correlation between serum miR-21 levels and GC prognosis

**Figure 1:**
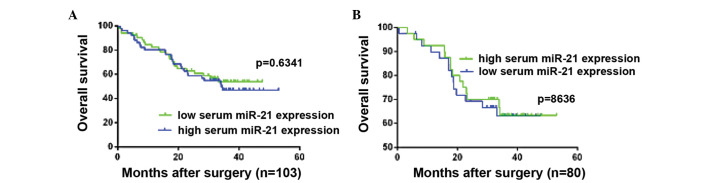
Kaplan-Meier overall survival curves for (A) all 103 gastric cancer (GC) patients, and (B) 80 GC patients who underwent curative surgery, according to the level of microRNA-21 (miR-21) expression. Log-rank tests showed that there were no significant differences between the low miR-21 expression and the high miR-21 expression groups (P=0.634 and P=0.863, respectively).

To evaluate whether the serum miR-21 level was associated with prognosis in patients with GC, a survival analysis was performed. The patients were divided into high miR-21 expression (n=51) and low miR-21 expression (n=52) groups. This division was based on the cut-off value determined as the median level of log-transformed relative quantity (−0.64) for the serum miR-21 expression levels. According to the Kaplan-Meier method, survival curves were not significantly different between the two groups (P=0.6341; Fig. 1A). A survival analysis for 80 of the 103 recruited patients who underwent radical surgery yielded the same results (P=0.8636; Fig. 1B).

### High serum miR-21 levels were associated with increased tumor size and advanced pT stage

**Figure 2:**
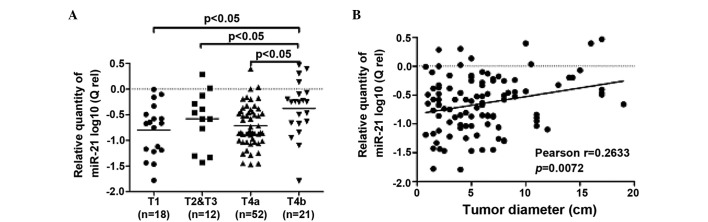
Serum microRNA-21 (miR-21) expression correlated with tumor size. (A) One-way ANOVA analyses showed that the serum miR-21 was significantly elevated in pT4b cases compared with pT1, pT2 and T3, and pT4a cases. There were no significant differences among pT1, pT2 and T3, and pT4a cases. (B) A Pearson correlation analysis showed that the serum miR-21 level and tumor size were positively correlated (r=0.2633, P=0.0072). The relative quantity (Qrel) of serum miR-21 was normalized to the serum miR-16 and miR-93 levels and expressed as the log_10_ of Qrel. The horizontal line indicates the mean.

In order to better understand the potential roles of serum miR-21 in GC development and progression, the correlation between the serum miR-21 levels and the clinicopathological factors of the GC patients were also assessed. The Mann-Whitney test showed that there was no marked correlation between the miR-21 levels and factors such as age, gender, differentiation, lymph node metastasis and TNM stage (Table I). However, the serum miR-21 level was significantly elevated in the pT4b cases compared with the pT1, pT2 and T3, and pT4a cases (P<0.05; Fig. 2A). Additionally, a Pearson correlation analysis showed that the serum miR-21 levels rose significantly when the tumor size was increased (r=0.2633, P=0.0072; Fig. 2B).

### Cox analyses of clinicopathological factors in GC patients

A univariate analysis showed that the tumor size, depth of invasion, lymph node metastasis, TNM stages and surgical method were significantly correlated with post-operative survival. The multivariate Cox proportional hazard regression analysis indicated that TNM stage and surgical method were significantly independent prognostic factors for patients with GC (P<0.05 and P=0.024, respectively; Table II).

## Discussion

In the present study, serum miR-21 was examined in order to explore its potential role as a prognostic biomarker for patients with GC. The findings showed that serum miR-21 levels were not able to predict a prognosis in the patients with GC. The serum miR-21 expression levels were positively correlated with tumor size, indicating that patients with higher serum miR-21 levels have larger tumors.

In contrast to studies with regard to miR-21 in tissues and cells of various types of cancer, research into miR-21 in the serum of GC patients is lacking. Serum miR-21 has been reported to be overexpressed in numerous types of cancer, but reduced levels have been observed after tumor resection (17,20–23). Therefore, it is thought that miR-21 may serve as a potential broad-spectrum serum-based biomarker for the diagnosis of certain solid tumors (24). Despite growing evidence highlighting its diagnostic value in various types of cancer, few studies have systematically explored the prognostic role of serum miR-21. To the best of our knowledge, the present study is the first to determine whether serum miR-21 levels may be used to predict a prognosis in patients with GC.

In the present study, the survival curves showed that there was no significant difference between the higher and lower serum miR-21 expression groups. The subgroup analysis was defined according to whether patients underwent radical surgery, and the same results were shown. Chan *et al*(12) and Ueda *et al*(14) reached the conclusion that tissue miR-21 did not affect the clinical prognosis of GC, consistent with the present results. Extensive research has revealed that miR-21 is involved in proliferation, the cell cycle, metastasis and the chemosensitivity of tumor cells by targeting several tumor suppressor genes, including PTEN, MARCKS, PDCD4 and Cdc25A (25–28). The correlation of tissue miR-21 expression levels and clinical stage, lymph node metastasis and prognosis of cancer patients were also evaluated (13,14). A correlation has rarely been reported between serum miR-21 expression levels and clinicopathological factors (12). This is likely due to the fact that the origin and biological function of circulating miRNAs is poorly understood. Recently, it was reported that miRNAs are selectively secreted into the circulation and may mediate intercellular communication (29,30). The miRNA expression profiles in tissues and cells are likely to be very different from those miRNAs in circulation. Similar miRNAs expressed in tissues and cells and those in circulation may play different biological roles in cancer development. Therefore, it is necessary to determine how serum miR-21 is involved in GC development. Clarifying the correlation between the serum miR-21 level and the clinicopathological factors in patients with GC may provide certain clues as to why serum miR-21 is not a predictor of GC prognosis.

The present study identified that higher serum miR-21 levels were associated with an increased tumor size and an advanced pT stage. Contradicting these results, Zheng *et al* identified that miR-21 levels in circulating tumor cells, not circulating miR-21 in serum, were associated with tumor size, TNM stages and tissue categories in GC patients (20). Based on the findings of the present study, we hypothesize that serum miR-21 levels may indirectly reflect the tumor burden in GC patients, with serum miR-21 expression reduced following effective treatment. There have been several reports stating that circulating miR-21 serum levels are significantly reduced after tumor resection (17,20–23). The measurement of serum miR-21 levels may have several promising clinical applications inpatients with GC, including confirming the completeness of the tumor resection, evaluating the efficacy of adjuvant therapies and monitoring disease recurrence during the follow-up period.

In the present study, a Cox hazard regression model analysis showed that the tumor size and depth of invasion were predictors of prognosis in the GC patients, but they were not independent factors. This may explain why the serum miR-21 levels were correlated with tumor size and depth of invasion, but were not able to predict prognosis. As expected, the TNM stages and surgical method were significant independent prognostic factors in the patient cohort.

In addition, only serum miR-16 (without internal references) has previously been used for quantifying serum miRNAs. In the present study, serum miR-16 and miR-93 were used as double internal references (19) when calculating the Qrel of serum miR-21 by qPCR (31), leading to more convincing and reliable qPCR data.

In conclusion, serum miR-21 levels were not able to predict the prognosis of patients with GC. However, it was associated with increased tumor size and advanced pT stage. The expression level of serum miR-21 may be an indicator for the tumor burden in GC patients, thereby making serum miR-21 a reliable biomarker for effective therapies, such as chemotherapy and surgical resection.

## Figures and Tables

**Table I:** Correlation between clinicopathological factors and expression of miR-21 in serum.

Clinicopathological factors	n	miR-21 expression (mean ± SD)	P-value
Gender			0.504
Male	68	−0.611±0.509	
Female	35	−0.705±0.434	
Age			0.712
≤60	53	−0.653±0.491	
>60	50	−0.631±0.483	
Tumor size			0.048^a^
≤5 cm	52	−0.713±0.507	
>5 cm	51	−0.571±0.455	
Tumor thickness			0.021^a^
pT1	18	−0.798±0.507	
pT2 + pT3	12	−0.577±0.547	
pT4a	52	−0.712±0.407	
pT4b	21	−0.375±0.531	
Nodal status			0.376
pN0	25	−0.778±0.543	
pN1	14	−0.592±0.521	
pN2	17	−0.526±0.462	
pN3	47	−0.628±0.447	
Distant metastasis			0.196
M0	89	−0.667±0.491	
M1	14	−0.486±0.425	
Venous invasion			0.750
Positive	28	−0.617±0.486	
Negative	75	−0.652±0.485	
Tumor differentiation			0.443
Poor	59	−0.611±0.452	
Moderate/well	44	−0.685±0.528	
UICC stage			0.238
I + II	31	−0.744±0.509	
III	58	−0.626±0.481	
IV	14	−0.486±0.425	
Surgery type			0.100
Radical	80	−0.690±0.470	
Palliative	23	−0.478±0.508	

a Indicates a significant difference (P<0.05).

Since the relative quantity (Qrel) was logarithmic, Qrel was transformed through logarithmic transformation into linear distribution for statistical convenience. The negative expression values indicated that miR-21 was expressed less than arbitrary samples. UICC, Union for International Cancer Control; SD, standard deviation.

**Table II:** Univariate and multivariate Cox analysis of prognostic factors in patients with GC.

		Univariate			Multivariate		
			
Factors	Categories	RR	RR (95% CI)	P-value	RR	RR (95% CI)	P-value
Gender	Male/female	0.763	0.429–1.335	0.356	-	-	-
Age	>60/≤60	0.975	0.557–1.708	0.975	-	-	-
Tumor size	≥5/<5	2.685	1.423–5.070	0.002^a^	0.995	0.471–2.10	0.989
Depth of invasion	T1 + T2/T3 + T4	5.960	2.140–16.60	0.001^a^	0.001	0.0001–9.40	0.941
Lymph node metastasis	Positive/negative	7.001	2.173–22.554	0.001^a^	2.066	0.306–13.92	0.456
Differentiation	Poor/moderate	1.686	0.936–3.039	0.082	-	-	-
Venous invasion	Positive/negative	1.562	0.859–2.842	0.144	-	-	-
TNM stage	I + II/IV	0.097	0.034–0.275	<0.0001^a^	0.143	0.047–0.435	0.001^a^
	III/IV	0.272	0.134–0.553	<0.0001^a^	0.401	0.177–0.905	0.028^a^
Surgery type	Palliative/radical	0.195	0.110–0.347	<0.0001^a^	0.458	0.232–0.904	0.024^a^
Serum miR-21 expression	High/low	0.873	0.498–1.530	0.873	-	-	-

a Indicates a significant difference (P<0.05).

CI, confidence interval; GC, gastric cancer.

## Abbreviations

GC: gastric cancer
miRNAs: microRNAs
miR-21: microRNA-21
